# Stent loaded with radioactive Iodine-125 seeds for adenoid cystic carcinoma of central airway: A case report of innovative brachytherapy

**DOI:** 10.3389/fonc.2023.837394

**Published:** 2023-03-28

**Authors:** Mingyao Ke, Junli Zeng, Zhide Chen, Rui Huang, Xuemei Wu, Shuyuan Chu

**Affiliations:** ^1^ Department of Respiratory Centre, The Second Affiliated Hospital of Xiamen Medical College, Xiamen, Fujian, China; ^2^ Laboratory of Respiratory Disease, Affiliated Hospital of Guilin Medical University, Guilin, Guangxi, China

**Keywords:** adenoid cystic carcinoma, central airway, brachytherapy, radioactive stent, bronchoscope

## Abstract

Adenoid cystic carcinoma (ACC) of central airway is very rare. More than half of ACCs are unresectable for tumor extension. There’s rare report on local ACCs only in central airway. We present a case of ACC in central airway who underwent an innovative brachytherapy. A 44-year-old woman was diagnosed with primary ACC in central airway without regional lymphadenopathy or metastatic disease. Stenosis was observed in lower trachea and both left and right main bronchi (stenosis in lumen ≥50%) with bronchoscopy. The tumor was unresectable due to local extension. A Y-shaped and stainless-steel stent loaded with radioactive ^125^I seeds was placed in the central airway using bronchoscope. The number and distribution of ^125^I seeds were planed using treatment planning system. The stent was removed three months later. The patient tolerated the procedure well. She was alive without relapse three years after removing the stent with ^125^I seeds. This case demonstrates the successful use of stent with radioactive ^125^I seeds for unresectable ACCs in central airway. In the procedure, the stent was placed with bronchoscope and under the vision from bronchoscope. This innovative brachytherapy is well-tolerated, safe, precise and individualized designed. The patient with unresectable ACCs could get a long-term relapse-free survival. Clinical trials could be taken to validate its effectiveness and tolerability in patients with ACCs of central airway.

## Introduction

Malignant obstruction in central airway is usually involved with tumors in trachea or mainstem bronchi. In general, tracheal tumors are uncommon, making up only 0.2% of all respiratory malignancies (1). Adenoid cystic carcinoma (ACC) of trachea accounts for approximately 15%-20% of primary tracheal carcinomas (2). Thus, there’s rare report on local ACC only in central airway. The ACC in central airway poses a challenge to diagnosis and treatment. Here, we present a rare case of unresectable ACC in central airway, who underwent an innovative brachytherapy. She was placed a stainless-steel stent loaded with radioactive Iodine-125 (^125^I) seeds in central airway using bronchoscope under the guidance from bronchoscope. At the most recent follow-up, she achieved a three-year relapse-free survival after removing the stent with ^125^I seeds.

## Case description

A 44-year-old woman presented with cough for one year and bloody phlegm for six months. She also presented with shortness of breath after walking on a level road for three months. The bronchoscopic biopsy confirmed a diagnosis of primary ACC in her central airway. The work-up with positron emission tomography and computed tomography (PET-CT) showed negative for regional lymphadenopathy and metastatic disease. However, the ACC was unresectable due to the extension of tumor.

**Figure 1:**
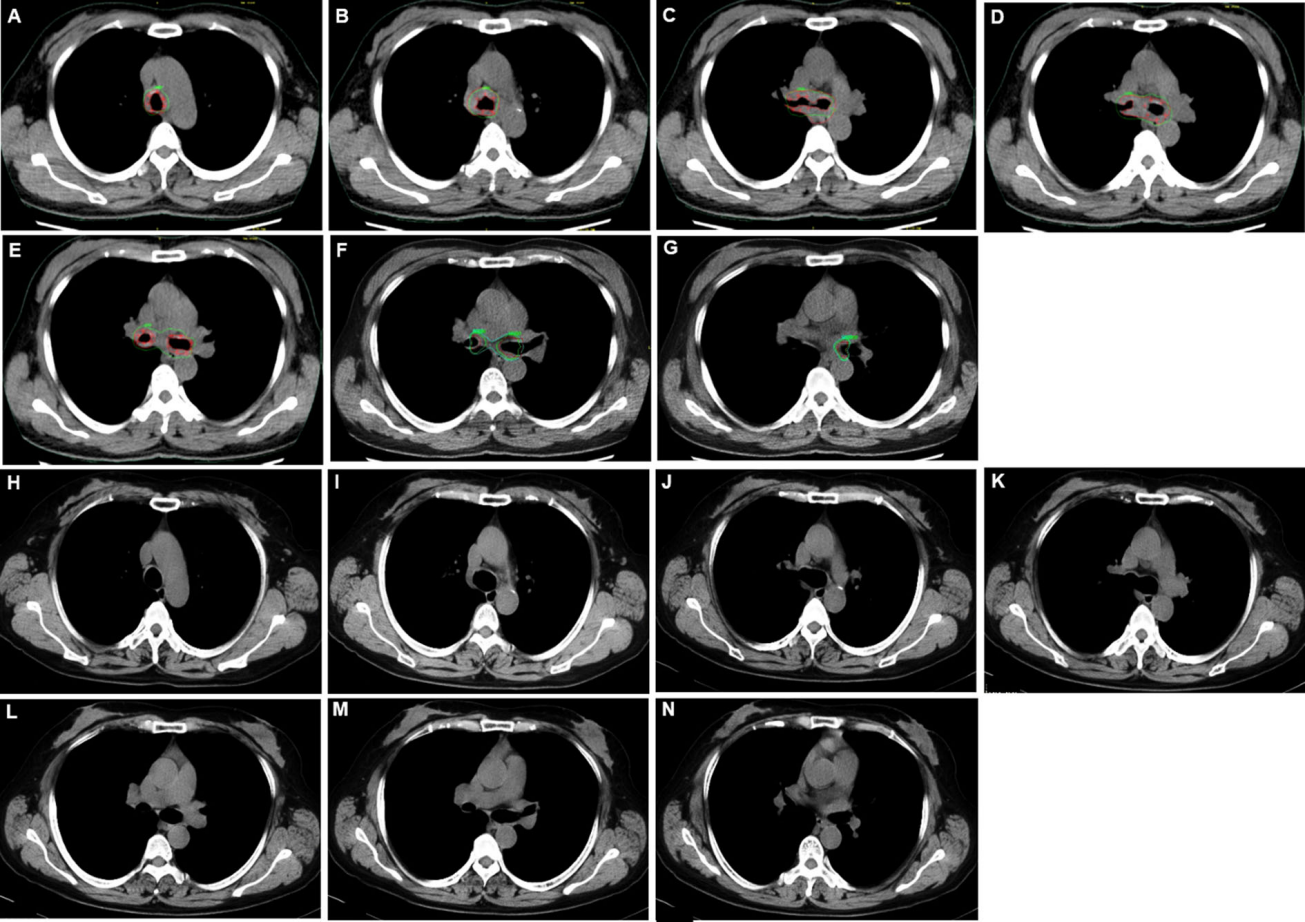
Thoracic computerized tomography (CT) scans for adenoid cystic carcinoma (ACC) in central airway. **(A–G)** Thoracic CT scans before placing the stent with ^125^I seeds. **(H–N)** Thoracic CT scans 35 months after removing the stent with ^125^I seeds.

**Figure 2:**
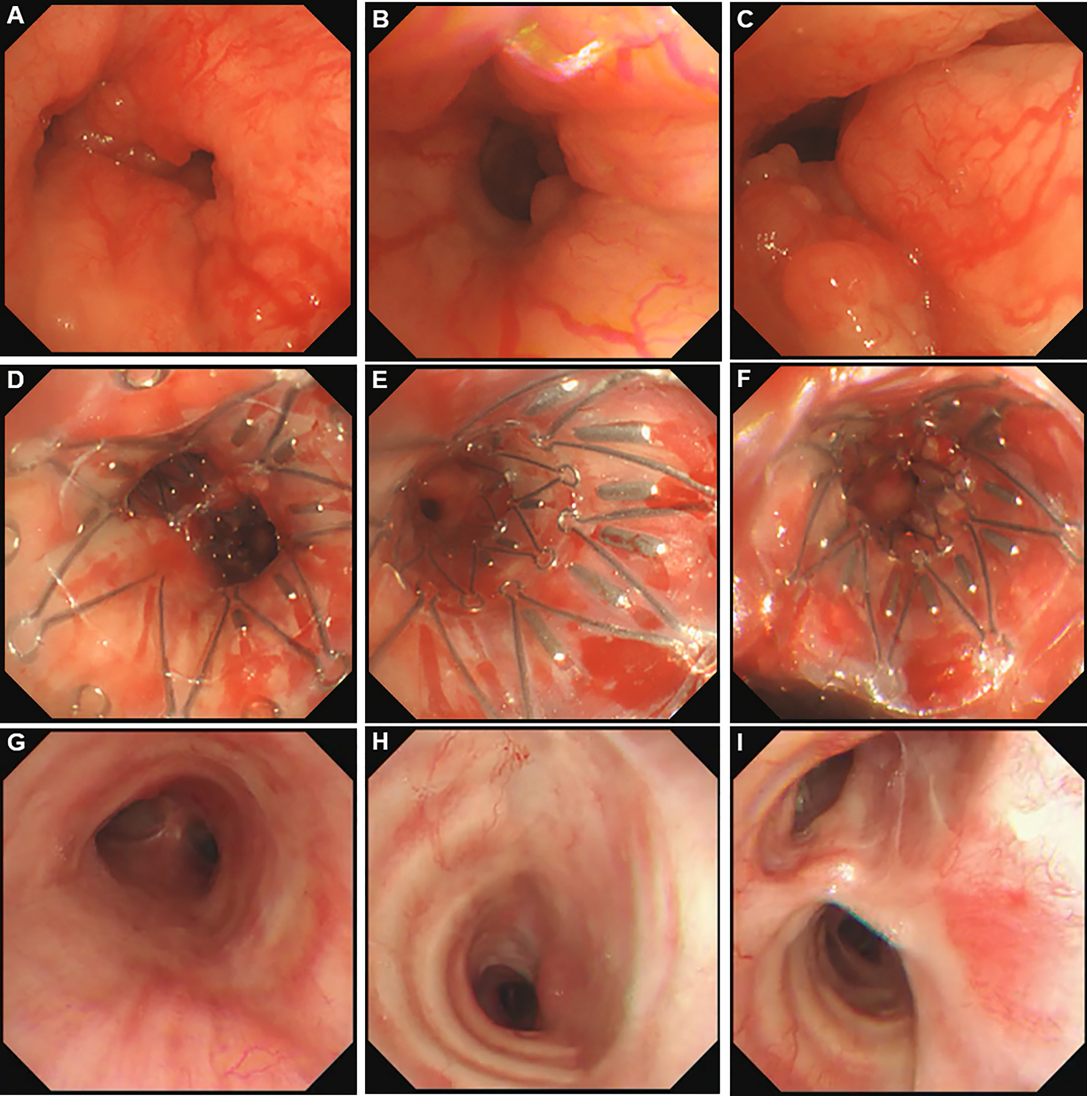
Bronchoscopy pictures in central airway. **(A)** Adenoid cystic carcinoma (ACC) in lower trachea. **(B)** ACC in left main bronchus. **(C)** ACC in right main bronchus. **(D)** Stent carrying ^125^I seeds in lower trachea. **(E)** Stent carrying ^125^I seeds in left main bronchus. **(F)** Stent carrying ^125^I seeds in right main bronchus. **(G)** lower trachea 35 months after removing the stent. **(H)** Left main bronchus 35 months after removing the stent. **(I)** Right main bronchus 35 months after removing the stent.

She was referred to our hospital. In physical examination, the intensity of her breath sounds in both lungs was decreased. Thoracic computerized tomography (CT) scanning showed soft tissue nodules at the bifurcation of the right main bronchus (**Figures 1A–G**). On July 12, 2018, bronchoscopy showed swelling and congestion of mucosa in lower trachea, widened and rough carina, and stenosis in lower trachea and both left and right main bronchi (stenosis in lumen ≥50%) (**Figures 2A–C**). With bronchoscope, she underwent ablative therapy to remove visible tumors using high-frequency electrocautery system, as well as balloon dilation for stenosis in central airway.

**Figure 3:**
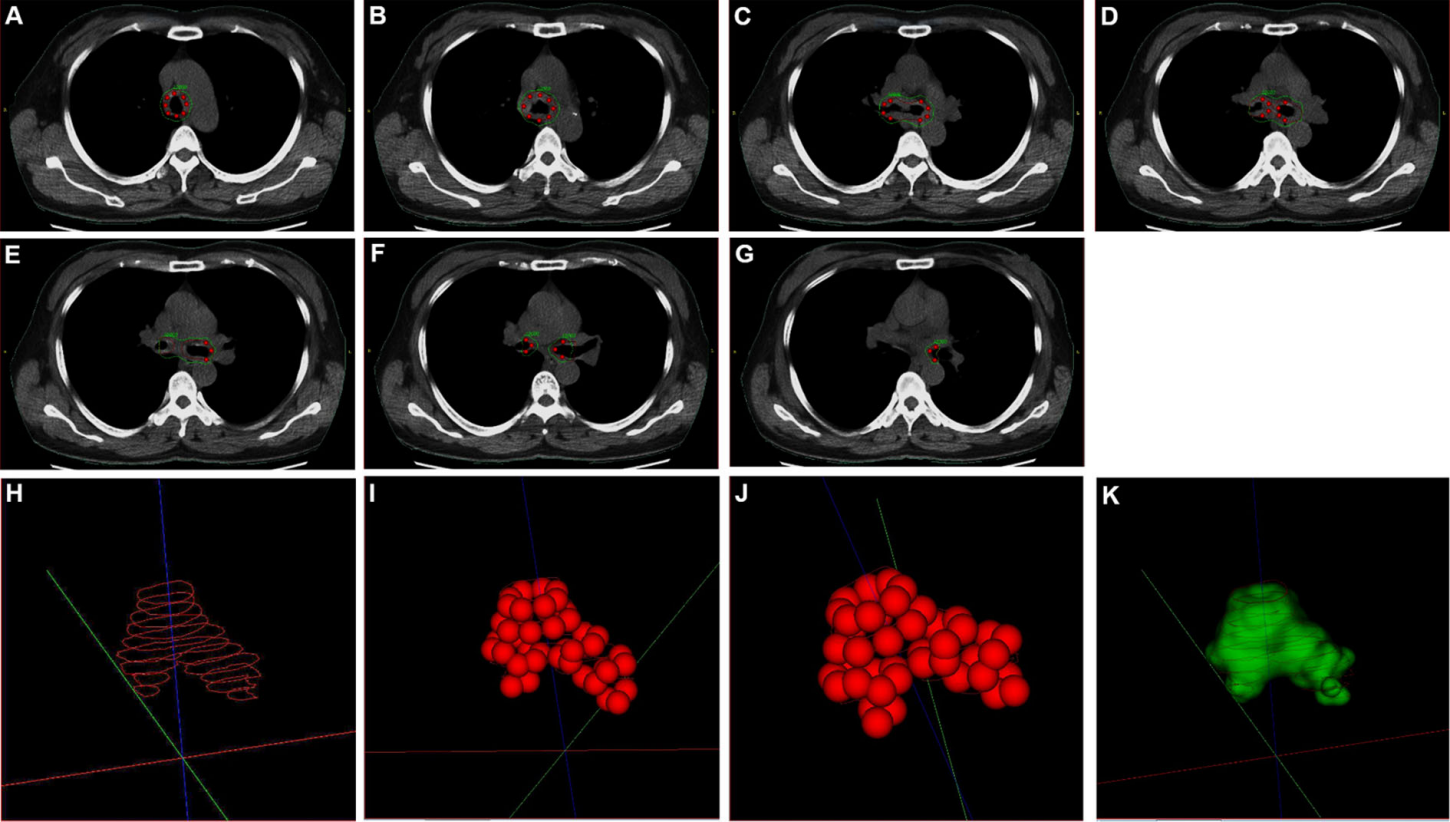
The number and distribution of seeds in plan before placement. **(A–G)** The seeds distribution and isodose distribution in thoracic computerized tomography (CT) scans. **(H)** Planning target volume (PTV) in three-dimension (3D). **(I, J)** ^125^I seeds distribution in 3D. **(K)** Isodose cloud of 120Gy in 3D. Red seeds: ^125^I seeds. Red curve: target area in planning. Green curve: Isodose curve of 120Gy.

On July 24, 2018, under the guidance from bronchoscope, a Y-shaped and stainless-steel stent loaded with 40 radioactive ^125^I seeds (0.60 mCi) was placed in her lower trachea using bronchoscope (**Figures 2D–F**; **Supplementary File 1**). The number and distribution of seeds were planed using treatment planning system (TPS) (Beijing Feitianzhaoye Co., Ltd.) (**Figure 3**) before placement based on the thoracic CT scanning (**Figures 1A–G**). A dose of 120Gy was prescribed to the planning target volume (PTV). All layers of trachea and mainstem bronchi with the tumor in CT scans were contoured as clinical target volume (CTV), because the depth of tumor invasion can’t be determined from CT scans. Moreover, the stent with ^125^I seeds was placed in the central airway. And the distance from tumor to the seeds remained the same even when breathing or changing body positions. Thus, PTV wasn’t extended from CTV. For organs at risk, only spinal cord was contoured, but wasn’t included in PTV. The dose for spinal cord at risk was constrained less than 45Gy, which was referenced from the dose of external beam radiotherapy.

For radiation protection, the patient, endoscopist and assistant wore lead apron in the procedure. The lead apron covered both front and back of the body. In a lead box with a lead-glass window, the stent was loaded with radioactive ^125^I seeds, and then was put into a metal and radiation-resistant stent-conveyor. During this process, the endoscopist was wearing lead gloves. Thus, the endoscopist and assistant were protected from ^125^I radiation in the procedure. The patient tolerated the procedure well without chest pain, fistula formation, pulmonary infection, pneumothorax, hemoptysis or stent displacement. No bleeding was observed at the site. When she was discharged home, the radioactive background of the patient was the same as that from the ^125^I seeds. The background was nearly zero when she’s wearing lead apron. The patient was given precaution instructions which were similar to other low-dose-rate (LDR) brachytherapy. Particularly, she had to wear the lead apron whenever she was in a room with any other person till the stent with ^125^I seeds was removed. This lead apron covered both front and back of her body. We required her family to help her keep doing this. We also followed her every month to make sure she did it till removing the stent with ^125^I seeds.

For this case, the stent with radioactive ^125^I seeds was kept in the stenosis for about 1.5 half-lives of ^125^I seeds. The reason was that the radioactive activity of ^125^I reduces to 25% after two half-lives. While this duration was necessary to deliver the majority of the activity, there were potential side effects due to the stent, such as septum excretion. Thus, we proposed that the stent should be kept in the airway for one to two half-lives of ^125^I seeds. On October 15, 2018, the Y-shaped stent with radioactive ^125^I seeds was removed using bronchoscope. No visible airway stenosis was observed in bronchoscopy. Tumor cells were not found with bronchoscopic brushing. At that time, she didn’t present with shortness of breath or bloody phlegm. The patient was followed with thoracic CT scanning and bronchoscopy. At the most recent follow-up on September 9, 2021, the patient was alive without symptoms, and her central airway looked like normal without stenosis or tumors (**Figures 1H–N**, **2G–I**; **Supplementary File 2**). To the best of our knowledge, no case of ACC in central airway has been reported to be treated with stent loaded with radioactive ^125^I seeds using bronchoscope with the guidance of bronchoscope, and then got three-year relapse-free survival. **Table 1** illustrates the timeline with the episode of care. The authors are accountable for all aspects of the work in ensuring that questions related to the accuracy or integrity of any part of the work are appropriately investigated and resolved. All procedures performed in the study involving human participant were in accordance with the ethical standards of the institutional research committee and with the Helsinki Declaration (as revised in 2013). Written informed consent was obtained from the patient for publication of this case report and accompanying images.

**Table 1:** The timeline with the episode of care.

Date	Episode of care
July 12, 2018	1. Bronchoscopy showed stenosis in central airway. 2. The patient underwent bronchoscopic ablative therapy and balloon dilation.
July 24, 2018	A Y-shaped and stainless-steel stent loaded with radioactive ^125^I seeds was placed in the lower trachea with bronchoscope and under the vision from bronchoscope.
October 15, 2018	The Y-shaped stent with radioactive ^125^I seeds was removed using bronchoscope.
September 9, 2021	In the most recent follow-up, the patient was alive without symptoms, and her central airway was almost normal without stenosis or tumors.

## Discussion

ACC is a slow-growing and painless tumor, which has a potential to invade into surrounding tissues. It’s preferred to remove the entire tumor by a wide surgical excision. However, ACCs usually have involved a long segment of airway before it’s obstructive. Thus, most of them are unresectable when patients present symptoms. In the experience of Massachusetts General Hospital, 25% of patients with ACCs had unresectable tumor (1). As determined by bronchoscopy, tumor length was the most common reason that resection was declined in 68% of these patients (1). Our case was one of those 68% patients. Mostly, neither signs nor symptoms of ACCs are found for many months and years before they’re diagnosed (1). For our case, when the ACC was diagnosed, the tumor had already been unresectable. At this time, local therapy was an effective alternative to provide meaningful palliation and good life quality to the patient. Thus, brachytherapy should be an appropriate option for this patient.

Stent loaded with radioactive seeds has been widely used for malignant airway obstruction due to lung cancer or esophageal cancer (3, 4), which is under guidance of CT or C-arm angiographic unit. In contrast, we placed the stent with radioactive ^125^I seeds under the vision from bronchoscope in this patient with ACC of central airway. This innovative brachytherapy hasn’t been reported yet. Compared with CT-guided percutaneous placement, this innovative one could accurately place the stent in the stenotic segment of airway under guidance of bronchoscope, without side effect such as chest pain, fistula formation or pulmonary infection. Moreover, both patient and doctors exposed less radiation in the process of this innovative brachytherapy than in the CT-guided one. For ACC in airway, the tumor cells usually infiltrate into the wall of airway. With the innovative brachytherapy, the stent could keep the radioactive seeds exactly in the stenotic segment of airway. At the end of therapeutic period, the radioactive stent could be taken out using bronchoscope. All of those for treating ACC in airway can’t be done by percutaneous CT-guided radioactive stent placement.

In addition, the innovative brachytherapy could be better than traditional brachytherapy or after-loading radiation therapy for ACCs in central airway. The reasons are as follows. First, radioactive stent in airway could keep continuous brachytherapy with a therapeutic dose for the tumor in airway stenosis. This radioactive dose is too low to cause side effects which are happened in traditional brachytherapy. The ^125^I seed has a half-life of 59.4 days. The radioactive stent could be kept in the airway affected by tumor without displacement during the period of brachytherapy, which is one to two half-lives of ^125^I seed (5). For our case, the stent with radioactive ^125^I seeds was kept in the segment affected by tumor for about 1.5 half-lives of ^125^I seed. Second, the individualized dose and distribution of radioactive seeds are planned by TPS before placing into airway. That leads to more precise dose and location of seeds for the tumor and then a better effect than traditional brachytherapy, whereas less side effects. For our case, since the ACC had infiltrated into the tracheal and bronchial wall, it’s impossible to distinguish the tumor and normal tissues in airway wall even in contrast-enhanced CT scans. Thus, all layers of tracheal and bronchial wall were regarded as the PTV in our case with non-contrast CT pictures. It’s reported that a case of primary tracheal ACC was treated with high dose-rate brachytherapy, who developed tracheal stenosis 22 months after brachytherapy and had to be placed a tracheal stent (6). In contrast, our case wasn’t observed tracheal stenosis in the most recent follow-up, confirming the efficacy and safety of the innovative brachytherapy in the long term. Moreover, the procedure of this innovative brachytherapy is minimally invasive and well tolerated.

Tracheal ACCs were reported to be treated with a high dose of permanent Palladium-103 seed implantation under CT guidance (7). The three patients were followed for an average time of nine months, when they showed disease regression and symptom improvement. However, long-term results weren’t reported. In contrast, our case achieved a three-year relapse-free survival using ^125^I seeds in a low-dose irradiation. For our case, almost all of the ACC was eliminated by impermanently placing a stent with radioactive ^125^I seeds under the vision from bronchoscope. The ACC in central airway hasn’t been relapsed till the most recent follow-up, as long as 35 months after removing the stent with ^125^I seeds. The efficacy of this brachytherapy for our case suggests that ACCs should be radiosensitive for endobronchial ^125^I brachytherapy. Our finding is consistent with the previous report that tracheal tissue is radio-sensitivity (8). And radiotherapy is suggested as adjuvant therapy for primary tracheal tumors, particularly if the tumor is unresectable (8). That could support the endobronchial brachytherapy for ACCs in central airway. Moreover, it’s recently reported that ACCs in eye, head or neck were all radiosensitive for ^125^I brachytherapy (9, 10). That further confirms the radio-sensitivity of ACCs for ^125^I seeds in our case. The ^125^I seeds provide continuous radiation in a low dose, which could lead to a buildup of radiation damage through synchronizing tumor cells to radiosensitive G2-M phase (11). As a source of low-dose radiation, ^125^I seeds allow normal tissue to repair the sublethal damage, whereas the tumor cells are damaged and killed (12). Therefore, ^125^I seeds could be appropriate in brachytherapy for ACCs in airway.

For organs at risk, only spinal cord was contoured for our case. The reason is as follows. The distance from spinal cord to the nearest ^125^I seeds was about 1cm. The radiative dose is sharply attenuated at a distance more than 1cm. Therefore, the dose of irradiation is little and safe for organs such as esophagus, heart or spinal cord in LDR brachytherapy. Even in high-dose-rate endobronchial brachytherapy or interstitial brachytherapy, the dose exposed in spinal cord, heart and esophagus is safe (13, 14), let alone LDR brachytherapy for our case. Spinal cord was contoured in our case, because it’s so crucial that any overdose could lead to irreversible injury. For our case, a dose of 45Gy was the limit for spinal cord at risk, which was referenced from the dose of external beam radiotherapy using conventional fractionation with 1.8-2Gy per fraction. That’s because there isn’t guideline of LDR brachytherapy for endobronchial cancer (15). Actually, the dose exposed in spinal cord was far less than 45Gy in practice.

Despite a slow-growing growth pattern, the National Cancer Institute (USA) considers ACC as a high-grade malignancy (16). According to previous report, for patients with tracheal ACC, the 5-year survival ranged from 33% to 52% and the 10-year survival ranged from 10% to 29% regardless of whether ACC was resected or not (17). Considering that, our innovative technique could be an appropriate option for unresectable ACCs in central airway. This innovative technique is an impermanent implantation of radioactive seeds using bronchoscope. It could provide a high quality of life to those patients with unresectable ACCs of central airway in the long term. However, we acknowledged the limitation of this innovative technique that it’s not appropriate to the tumor in peripheral lung.

In conclusion, this case demonstrates the successful use of stent loaded with radioactive ^125^I seeds for unresectable ACCs of central airway. In the procedure, the stent was placed with bronchoscope and under the vision from bronchoscope. This innovative brachytherapy is well-tolerated, safe and individualized designed, which could get a long-term relapse-free survival for patients with unresectable ACCs of central airway. Clinical trials could be taken to validate its effectiveness and tolerability in patients with ACCs and obstruction in central airway.

## Concluding remarks

Our case is the first time to demonstrate the successful use of stent with radioactive Iodine-125 seeds in unresectable ACCs of central airway, in which the stent was placed with bronchoscope and under the vision from bronchoscope. This innovative brachytherapy is well-tolerated, safe, precise and individualized designed, which could get a long-term relapse-free survival for patients. Considering the challenge of treatment for ACC in central airway, our findings may provide an innovative brachytherapy to treat the malignant airway obstruction.

## Data availability statement

The original contributions presented in the study are included in the article/**Supplementary Material**. Further inquiries can be directed to the corresponding authors.

## Ethics statement

Ethical review and approval was not required for the study on human participants in accordance with the local legislation and institutional requirements. The patient/participant provided her written informed consent to participate in this study. Written informed consent was obtained from the individual for the publication of any potentially identifiable images or data included in this article.

## Author contributions

MK, JZ, ZC, RH, and XW were responsible for the patient’s treatment and collected the patient’s information. SC drafted this manuscript. MK and SC revised the paper. MK, XW and SC offered constructive suggestions for this study. All authors contributed to the article and approved the submitted version.

## Supplementary material

Supplementary file 1 The video of bronchoscopy when the stent with Iodine-125 seeds was placed.

Supplementary file 2 The video of bronchoscopy 35 months after removing the stent with Iodine-125 seeds.
